# Using observed incidence to calibrate the transmission level of a mathematical model for *Plasmodium vivax* dynamics including case management and importation

**DOI:** 10.1016/j.mbs.2021.108750

**Published:** 2022-01

**Authors:** Clara Champagne, Maximilian Gerhards, Justin Lana, Bernardo García Espinosa, Christina Bradley, Oscar González, Justin M. Cohen, Arnaud Le Menach, Michael T. White, Emilie Pothin

**Affiliations:** a Swiss Tropical and Public Health Institute, Socinstrasse 57, P.O. Box, Basel, CH-4002, Switzerland; b University of Basel, Petersplatz 1, P.O. Box, Basel, CH-4001, Switzerland; c Clinton Health Access Initiative, 383 Dorchester Ave, Suite 400, Boston, 02127, MA, USA; d Ministerio de Salud de Panama, Calle culebra, Edificio 265 del Ministerio de Salud, Corregimiento de Ancón, Panama; e Institut Pasteur, Université de Paris, G5 Épidémiologie et Analyse des Maladies Infectieuses, Département de Santé Globale, Paris, F-75015, France

**Keywords:** Plasmodium vivax model, Malaria model, Panama, Plasmodium vivax radical cure

## Abstract

In this work, we present a simple and flexible model for *Plasmodium vivax* dynamics which can be easily combined with routinely collected data on local and imported case counts to quantify transmission intensity and simulate control strategies. This model extends the model from White et al. (2016) by including case management interventions targeting liver-stage or blood-stage parasites, as well as imported infections. The endemic steady state of the model is used to derive a relationship between the observed incidence and the transmission rate in order to calculate reproduction numbers and simulate intervention scenarios. To illustrate its potential applications, the model is used to calculate local reproduction numbers in Panama and identify areas of sustained malaria transmission that should be targeted by control interventions.

## Highlights

• A model for *P. vivax* including case management and imported infections is analysed.
• The model is calibrated by linking its steady state to observed incidence data.
• This method can be used to calculate reproduction numbers or simulate interventions.
• Parameter sensitivity and coherence with an existing model are explored.
• Practical applications of the model are illustrated in the Panama context.

## 1 Introduction

Long considered as a benign malaria parasite, *Plasmodium vivax* is now increasingly recognized as an important public health issue, due to its potential severe clinical outcomes, its large health and socio-economic burden and the challenges associated with its elimination and control [1], [2], [3]. In 2017, [4] estimated about 14 million cases worldwide, and more than 3 billion people living within the limits of the parasite transmission across the globe. A distinguishing characteristic of *P. vivax* is its capacity to remain dormant and undetected in the liver of the infected host for long periods of time. The parasite reservoir in the liver stage is still difficult to measure and quantify (even though there are recent advances [5]), but its reactivation, i.e. relapse infections, are believed to constitute a large fraction of reported incident infections [6]. This mechanism, along with other specific characteristics such as the lower density infections that are harder to detect, increases parasite robustness in a wide range of environments [2], [7] and thus complicates elimination efforts. As a result, while *P. falciparum* elimination is well advanced in Central America and the Greater Mekong Subregion, *P. vivax* incidence reductions have been slower. As such, drugs to clear liver stage in addition to blood stage parasites are required to avoid reinfections and potentially reduce transmission, though the drugs currently existing to clear liver-stage parasites, 8-aminoquinolines such as primaquine (PQ) and tafenoquine (TQ), are difficult to implement [2].

In this context, mathematical modelling can prove useful to help disentangle the effects of these *P. vivax* specificities and explore the potential effectiveness of different control interventions [2]. Several population-level mechanistic *P. vivax* models were introduced over the last decades to explicitly include the relapse mechanism [7], [8], [9], [10], [11], [12], [13], [14] and they have been used to explore the effect of interventions such as treatment, bednet distributions or mass drug administration [6], [10], [11], [12], [13], [14], [15]. Among the models that include treatment, some focus exclusively on liver stage treatment [12] while in others the blood stage treatment is also added, either as a reduced clearance rate assumed for all individuals [13] or as a specific disease state [14]. Some *P. vivax* models also simulate the effect of the importation of infections from one area to another, by explicitly representing the movement of individuals between regions or countries [14], [16].

Furthermore, mathematical models are useful tools to support the strategic planning of country, district or local malaria control measures [17]. In particular, mathematical models can be used to simulate the effect of differing intervention scenarios, such as changes in case management or vector control practices, and hence explore where and under what conditions these interventions would be most impactful [15], [18]. For these applications, it is necessary to first calibrate the model to the transmission intensity level of each considered setting, and this step can prove challenging for complex models. In addition, imported cases should be taken into account during this calibration step in order to distinguish the areas with sustained transmission from those where transmission is “importation-driven” [19] i.e. vulnerable areas where the disease would disappear in the absence of importation.

While many models for *P. vivax* already exist, they are not readily operationalized for being used routinely at country level, either because they do not include all these features, or because their calibration is not straightforward to implement. Therefore, in this work, we present a simple and flexible *P. vivax* model that makes use of routinely collected local scale case count, intervention and importation data to quantify transmission intensity and simulate control strategies. This model is derived from [7] and extended to include case management interventions and imported infections. It has the potential to include vector control as well in future applications. The endemic steady state of the model is used to derive a relationship between the observed incidence and the transmission rate, and thus to calculate reproduction numbers and simulate intervention scenarios based on routine data at local scales.

The remaining sections are structured as follows: In Section 2, the *P. vivax* transmission model is presented and the associated reproduction numbers are derived. The methodology to calculate the transmission rate from incidence and importation data is presented in Section 3. A sensitivity analysis to evaluate the effect of the parameters on model outcomes (reproduction numbers and proportion of relapses) in presented in Section 4. To evaluate the model’s coherency with previous published literature, in Section 5, the effect sizes for an intervention change predicted by the model are compared to the ones from [15]. Section 6 presents an application of the model: the local level transmission potential of *P. vivax* in 2018 in Panama is assessed using malaria case reports. Finally, Section 7 discusses the results and concludes.

## 2 A compartmental model for *P. vivax* dynamics accounting for case management and importation

### 2.1 Model description

**Fig. 1:**
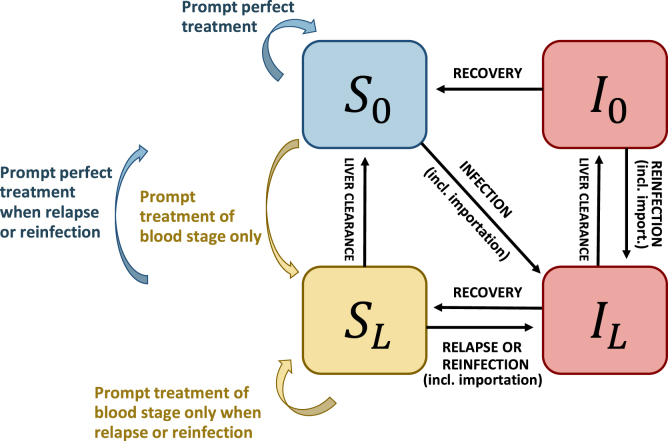
Schematic representation of the model, derived from [7]. The state variables $S_{0}$, $S_{L}$, $I_{0}$ and $I_{L}$ are defined in the text and in Table 1. Red indicates infectious individuals with blood-stage infection, yellow indicates non-infectious individuals with latent liver-stage parasites and blue indicates susceptible malaria-free individuals.

*P. vivax* dynamics are represented within a deterministic compartmental model derived from [7] (tropical model version). The original model was simplified by removing the equations representing the vector dynamics, thus this model approximates the transmission process with inter-human transmission similar to classical SIS models. The model is described by a system of ordinary differential equations schematically presented in Fig. 1, where $I_{L}$ is the proportion of infectious individuals with liver and blood stage parasites, $I_{0}$ is the proportion of infectious individuals with only blood stage parasites, $S_{L}$ the proportion of susceptibles with liver stage parasites and $S_{0}$ the proportion of fully susceptible individuals, such that $I_{L}+I_{0}+S_{L}+S_{0}=1$. λ is the transmission rate, r is the blood-stage clearance rate, $\gamma_{L}$ is the liver-stage clearance rate and f is the relapse frequency (all parameters and state variables are presented in Table 1).

The model from [7] is extended in two ways. Firstly, importation is included as a rate δ, such that individuals can become infectious from a source other than the pool of infectious individuals in the study population, most likely because they are infected in another area although the explicit movement of people is not modelled.

Secondly, case management is included as a proportion of infections that would be cured before entering the infected compartments, and therefore these infections would not contribute to parasite transmission. The treatment success probability accounts for the blood and liver stages and is therefore described by two parameters. The effective treatment rate α represents the proportion of infections that would receive timely treatment and cure their blood stage parasites. The probability of radical cure β represents the proportion of treated individuals whose liver-stage parasites would be cleared. Therefore, among the new infections, a proportion αβ would be cured from both their blood and liver stage parasites, a proportion α(1−β) would be cured from their blood stage infection only, and a proportion 1−α would not be effectively treated. Additionally, it is assumed that individuals with a blood stage-only infection that are re-infected (and therefore develop an additional liver stage infection) do not seek care for the additional infection and therefore are not treated. The model is described by the following set of ordinary differential equations:

(1) $$\frac{dI_{L}}{dt}=\left(1-\alpha \right)\left(\lambda \left(I_{L}+I_{0}\right)+\delta \right)\left(S_{0}+S_{L}\right)\;+\;\left(\lambda \left(I_{L}+I_{0}\right)+\delta \right)I_{0}+\left(1-\alpha \right)fS_{L}-\gamma_{L}I_{L}-rI_{L}$$

(2) $$\frac{dI_{0}}{dt}=-\left(\lambda \left(I_{L}+I_{0}\right)+\delta \right)I_{0}+\gamma_{L}I_{L}-rI_{0}$$

(3) $$\frac{dS_{L}}{dt}=-\left(1-\alpha \left(1-\beta \right)\right)\left(\lambda \left(I_{L}+I_{0}\right)+\delta +f\right)S_{L}\;+\;\alpha \left(1-\beta \right)\left(\lambda \left(I_{0}+I_{L}\right)+\delta \right)S_{0}-\gamma_{L}S_{L}+rI_{L}$$

(4) $$\frac{dS_{0}}{dt}=-\left(1-\alpha \beta \right)\left(\lambda \left(I_{L}+I_{0}\right)+\delta \right)S_{0}+\left(\lambda \left(I_{0}+I_{L}\right)+\delta \right)\alpha \beta S_{L}\;+\;\alpha \beta fS_{L}+\gamma_{L}S_{L}+rI_{0}$$

Two special cases can be highlighted. When δ=0, the model ignores importation and reflects exclusively local transmission dynamics. The case where α=0 represents the transmission dynamics in the absence of treatment.

The model can easily be extended to include vector control as indicated in the concluding Section 7.

**Table 1:** Description of state variables and model parameters.

Notation	Description	Unit	Definition range
State variables			
$I_{L}$	Infectious individuals with liver and blood stage parasites	dimensionless	[0,1]
$I_{0}$	Infectious individuals with blood stage parasites only	dimensionless	[0,1]
$S_{L}$	Susceptibles individuals with liver stage parasites	dimensionless	[0,1]
$S_{0}$	Fully susceptible individuals	dimensionless	[0,1]

Parameters			
λ	Transmission rate	time^−1^	≥0
r	Blood stage clearance rate r	time^−1^	≥0
$\gamma_{L}$	Liver stage clearance rate	time^−1^	≥0
f	Relapse frequency	time^−1^	≥0
δ	Importation rate	time^−1^	≥0
α	Proportion of effective care	dimensionless	[0,1]
β	Proportion of radical cure	dimensionless	[0,1]
ρ	Observation rate	dimensionless	[0,1]

### 2.2 Reproduction numbers calculation

We define the reproduction numbers in the absence of importation (δ=0) to reflect the intrinsic transmission potential of a given setting. The basic reproduction number ($R_{0}$) is defined in the absence of control interventions (α=0) and the reproduction number under control ($R_{c}$) is defined in the presence of control interventions, i.e. the presence of treatment in the context of this model. The reproduction number in presence of control interventions is calculated using the next generation matrix method [20] with:

$$F=\left(\begin{array}{c} \left(1-\alpha \right)\lambda \left(I_{L}+I_{0}\right)S_{0}\\ 0\\ \alpha \left(1-\beta \right)\lambda \left(I_{L}+I_{0}\right)S_{0}\\ 0 \end{array}\right)$$

$$V=\left(\begin{array}{c} -\left(1-\alpha \right)\lambda \left(I_{L}+I_{0}\right)S_{L}-\lambda \left(I_{L}+I_{0}\right)I_{0}-\left(1-\alpha \right)fS_{L}+\gamma_{L}I_{L}+rI_{L}\\ \lambda \left(I_{L}+I_{0}\right)I_{0}-\gamma_{L}I_{L}+rI_{0}\\ \left(1-\alpha \left(1-\beta \right)\right)\lambda \left(I_{L}+I_{0}\right)S_{L}+\left(1-\alpha \left(1-\beta \right)\right)fS_{L}+\gamma_{L}S_{L}-rI_{L}\\ \left(1-\alpha \beta \right)\lambda \left(I_{L}+I_{0}\right)S_{0}-\alpha \beta \lambda \left(I_{0}+I_{L}\right)S_{L}-\alpha \beta fS_{L}-\gamma_{L}S_{L}-rI_{0} \end{array}\right)$$

The Jacobians in the disease-free equilibrium are:

$$DF\left(0,0,0,1\right)=\left(\begin{array}{cccc} \lambda \left(1-\alpha \right) & \lambda \left(1-\alpha \right) & 0 & 0\\ 0 & 0 & 0 & 0\\ \lambda \alpha \left(1-\beta \right) & \lambda \alpha \left(1-\beta \right) & 0 & 0\\ 0 & 0 & 0 & 0 \end{array}\right)$$

$$DV\left(0,0,0,1\right)=\left(\begin{array}{cccc} \gamma_{L}+r & 0 & -\left(1-\alpha \right)f & 0\\ -\gamma_{L} & r & 0 & 0\\ -r & 0 & \left(1-\alpha \left(1-\beta \right)\right)f+\gamma_{L} & 0\\ \left(1-\alpha \beta \right)\lambda & \left(1-\alpha \beta \right)\lambda -r & -\alpha \beta f-\gamma_{L} & 0 \end{array}\right)$$

And keeping only infected states we obtain:

$$F=\left(\begin{array}{ccc} \lambda \left(1-\alpha \right) & \lambda \left(1-\alpha \right) & 0\\ 0 & 0 & 0\\ \lambda \alpha \left(1-\beta \right) & \lambda \alpha \left(1-\beta \right) & 0 \end{array}\right)$$

$$V=\left(\begin{array}{ccc} \gamma_{L}+r & 0 & -\left(1-\alpha \right)f\\ -\gamma_{L} & r & 0\\ -r & 0 & \left(1-\alpha \left(1-\beta \right)\right)f+\gamma_{L} \end{array}\right)$$

The largest eigenvalue of $FV^{-1}$ is $R_{C}$. Using *SymPy* for symbolic calculation [21], we obtain:

(5) $$R_{C}=\frac{\lambda \left(1-\alpha \right)\left(\gamma_{L}+r\right)\left(f+\gamma_{L}\right)}{r\left[\gamma_{L}\left(f+\gamma_{L}+r\right)+\alpha f\left(\beta \left(r+\gamma_{L}\right)-\gamma_{L}\right)\right]}$$

Thus, the basic reproduction number is (setting α=0):

(6) $$R_{0}=\frac{\lambda \left(\gamma_{L}+f\right)\left(\gamma_{L}+r\right)}{r\gamma_{L}\left(f+\gamma_{L}+r\right)}=\frac{\lambda }{r}+\frac{\lambda f}{\gamma_{L}\left(f+\gamma_{L}+r\right)}$$

## 3 Calculation of the model transmission rate using data on incidence

This section indicates how to compute the transmission parameter λ from the observed incidence, using the equilibrium solution of the ODE model. The calculation accounts for the presence of both imported cases and ongoing control interventions and the λ estimate can then be plugged into (5), (6) to calculate setting specific $R_{c}$ and $R_{0}$. With this methodology, the reproduction numbers reflect the transmission potential of a given setting in the absence of importation if interventions are kept at their current level ($R_{c}$) or if interventions are removed ($R_{0}$).

### 3.1 Calculation of the transmission rate

We define $I≔I_{L}+I_{0}$ the proportion of blood-stage infections. Let $I_{L}^{*}$, $I_{0}^{*}$, $S_{L}^{*}$, $S_{0}^{*}$ and $I^{*}$ be the equilibrium proportions.

At the equilibrium, we have the equations

(7) $$0=\frac{dI_{L}}{dt}=\left(1-\alpha \right)\left(\lambda I^{*}+\delta \right)\left(1-I^{*}\right)+\left(\lambda I^{*}+\delta \right)I_{0}^{*}\;+\;\left(1-\alpha \right)fS_{L}^{*}-\gamma_{L}I_{L}^{*}-rI_{L}^{*}$$

(8) $$0=\frac{dI_{0}}{dt}=-\left(\lambda I^{*}+\delta \right)I_{0}^{*}+\gamma_{L}I_{L}^{*}-rI_{0}^{*}$$

(9) $$0=\frac{dS_{L}}{dt}=-\left(1-\alpha \left(1-\beta \right)\right)\left(\lambda I^{*}+\delta +f\right)S_{L}^{*}+\alpha \left(1-\beta \right)\left(\lambda I^{*}+\delta \right)S_{0}^{*}\;-\;\gamma_{L}S_{L}^{*}+rI_{L}^{*}$$

(10) $$0=\frac{dS_{0}}{dt}=-\left(1-\alpha \beta \right)\left(\lambda I^{*}+\delta \right)S_{0}^{*}+\left(\lambda I^{*}+\delta \right)\alpha \beta S_{L}^{*}+\alpha \beta fS_{L}^{*}\;+\;\gamma_{L}S_{L}^{*}+rI_{0}^{*}$$

By adding Eqs. (7), (8) we obtain the additional equation:

(11) $$0=\frac{dI}{dt}=\left(1-\alpha \right)\left(\lambda I^{*}+\delta \right)\left(1-I^{*}\right)+\left(1-\alpha \right)fS_{L}^{*}-rI^{*}$$

We define the observed incidence $h≔\rho \left[\left(\lambda I^{*}+\delta \right)\left(1-I^{*}\right)+fS_{L}^{*}\right]$ as the rate of observed newly arising blood-stage infections before treatment, where ρ is an observation rate. From the equilibrium equation for I, we obtain $h=\frac{r\rho I^{*}}{1-\alpha }$, and therefore $I^{*}$ can be derived from observed quantities and model parameters as:

(12) $$I^{*}=\frac{h\left(1-\alpha \right)}{\rho r}$$

As r>0 is necessary for the denominator to not be zero and because this condition is verified in all biologically plausible cases, we will continue with this assumption throughout the rest of the paper. If on the other hand h=0 or α=1, we have $I^{*}=0$. Being in the disease-free equilibrium makes it impossible to derive λ. Because of this, we will also make the two further assumptions that h>0 and α<1.

The proportion of imported cases p is defined such that $ph≔\rho \delta \left(1-I^{*}\right)$ represents the imported cases and $\left(1-p\right)h=\rho \left[\lambda I^{*}\left(1-I^{*}\right)+fS_{L}^{*}\right]$ the locally acquired cases. Therefore, δ can be derived from observed quantities and model parameters as:

(13) $$\delta =\frac{ph}{\rho \left(1-I^{*}\right)}=\frac{phr}{r\rho -h\left(1-\alpha \right)}$$

We then rely on the equilibrium relationships to calculate λ based on observed incidence h and the other model parameters. Solving (9) for $S_{L}^{*}$ by remembering that $S_{0}^{*}=1-I^{*}-S_{L}^{*}$ gives:

(14) $$S_{L}^{*}=\frac{rI_{L}^{*}+\alpha \left(1-\beta \right)\left(\lambda I^{*}+\delta \right)\left(1-I^{*}\right)}{\lambda I^{*}+\delta +\gamma_{L}+\left(1-\alpha \left(1-\beta \right)\right)f}$$

(if the denominator is 0 we necessarily have either $\lambda I^{*}=\delta =f=0$, which in turn yields h=0, or α=1, both of which we assumed not to be the case).

Likewise, we can solve Eq. (8) for $I_{L}^{*}$ by remembering that $I_{0}^{*}=I^{*}-I_{L}^{*}$:

(15) $$I_{L}^{*}=\frac{I^{*}\left[\lambda I^{*}+\delta +r\right]}{\lambda I^{*}+\delta +\gamma_{L}+r}$$

(the denominator is not zero as we assumed r>0).

Plugging (15) into (14) yields:

$$S_{L}^{*}=\frac{\alpha \left(1-\beta \right)\left(\lambda I^{*}+\delta \right)\left(1-I^{*}\right)\left(\lambda I^{*}+\delta +\gamma_{L}+r\right)+rI^{*}\left(\lambda I^{*}+\delta +r\right)}{\left(\lambda I^{*}+\delta +\gamma_{L}+\left(1-\alpha \left(1-\beta \right)\right)f\right)\left(\lambda I^{*}+\delta +\gamma_{L}+r\right)}$$

Now, plugging this into (11), multiplying by the denominator and dividing by (1−α) we obtain:

(16) $$0=\left(\lambda I^{*}+\delta +\gamma_{L}+\left(1-\alpha \left(1-\beta \right)\right)f\right)\left(\lambda I^{*}+\delta +\gamma_{L}+r\right)\times \;\left(\left(\lambda I^{*}+\delta \right)\left(1-I^{*}\right)-\frac{r}{1-\alpha }I^{*}\right)+\;f\alpha \left(1-\beta \right)\left(\lambda I^{*}+\delta \right)\left(1-I^{*}\right)\left(\lambda I^{*}+\delta +\gamma_{L}+r\right)+frI^{*}\left(\lambda I^{*}+\delta +r\right)$$

Rearranging the terms by powers of λ, we get the equation:

(17) $$0\;=\;\lambda^{3}{I^{*}}^{3}\left(1-I^{*}\right)+\lambda^{2}\left[{I^{*}}^{2}\left(1-I^{*}\right)\left(3\delta +2\gamma_{L}+r+f\right)-{I^{*}}^{3}\frac{r}{1-\alpha }\right]+\lambda \left[I^{*}\left(1-I^{*}\right)\left[\left(\delta +\gamma_{L}+f\right)\left(\delta +\gamma_{L}+r\right)+\delta \left(2\delta +2\gamma_{L}+r+f\right)\right]-{I^{*}}^{2}\frac{r}{1-\alpha }\left(2\delta +2\gamma_{L}+r+\alpha \beta f\right)\right]+\left(\delta +\gamma_{L}+r\right)\left(\delta \left(1-I^{*}\right)\left(\delta +\gamma_{L}+f\right)-\frac{rI^{*}}{1-\alpha }\left(\delta +\gamma_{L}+\left(1-\alpha \left(1-\beta \right)\right)f\right)\right)+fr\left(\delta +r\right)I^{*}$$

As long as the assumptions r>0, h>0 and α<1 are met, multiplication by the denominator of $S_{L}^{*}$ is an equivalent transformation, so any non-negative root of this polynomial is a solution of the system of equilibrium equations.

Even though there is an explicit formula for the roots of a polynomial of degree 3, the complexity of the coefficients makes it intractable. Nevertheless, we can analyse it for a qualitative result:

Theorem 1 *If* h>0 *,* r>0 *and* α<1 *, the function*

$$P\left(\lambda \right)=\lambda^{3}{I^{*}}^{3}\left(1-I^{*}\right)+\lambda^{2}\left[{I^{*}}^{2}\left(1-I^{*}\right)\left(3\delta +2\gamma_{L}+r+f\right)-{I^{*}}^{3}\frac{r}{1-\alpha }\right]+\lambda \left[I^{*}\left(1-I^{*}\right)\left[\left(\delta +\gamma_{L}+f\right)\left(\delta +\gamma_{L}+r\right)+\delta \left(2\delta +2\gamma_{L}+r+f\right)\right]-{I^{*}}^{2}\frac{r}{1-\alpha }\left(2\delta +2\gamma_{L}+r+\alpha \beta f\right)\right]+\left(\delta +\gamma_{L}+r\right)\left(\delta \left(1-I^{*}\right)\left(\delta +\gamma_{L}+f\right)-\frac{rI^{*}}{1-\alpha }\left(\delta +\gamma_{L}+\left(1-\alpha \left(1-\beta \right)\right)f\right)\right)+fr\left(\delta +r\right)I^{*}$$

*has at most one positive real root.*

*It has two non-negative real roots (i.e. one of them is* 0 *) only if* $\alpha \beta =\gamma_{L}=\delta =0$ *(corresponding to an equilibrium of relapses and recoveries without liver-stage clearance).*

The proof is given in A. This result makes it possible to calculate numerically the unique non-negative solution for given parameter values (except if $\alpha \beta =\gamma_{L}=\delta =0$).

### 3.2 Validity conditions

Theorem 1 guarantees that there cannot be more than one positive root, however some cases arise where there is no positive solution for λ. To illustrate these situations, we can note from the definition of h that:

$$\frac{h}{\rho }=\lambda I^{*}\left(1-I^{*}\right)+\delta \left(1-I^{*}\right)+fS_{L}^{*}\frac{h}{\rho }=\lambda I^{*}\left(1-I^{*}\right)+p\frac{h}{\rho }+fS_{L}^{*}\lambda I^{*}\left(1-I^{*}\right)=\frac{h}{\rho }-p\frac{h}{\rho }-fS_{L}^{*}$$

In realistic situations which are compatible with the endemic equilibrium, we have $0<I^{*}<1$, so we can deduce that if λ>0, then

(18) $$\frac{h}{\rho }>p\frac{h}{\rho }+fS_{L}^{*}$$

Because local vector-borne transmission cannot be negative (no biological meaning), the total of new infections due to relapses ($fS_{L}^{*}$) and importation ($p\frac{h}{\rho }$) should not exceed the total number of new infections ($\frac{h}{\rho }$). As $S_{L}^{*}$ depends on λ, Eq. (18) does not provide a criterion for the existence of a positive solution. The criterion on the parameters α, β, $\gamma_{L}$, δ, r and f as well as $I^{*}$ is presented in Theorem 2.

Theorem 2 *If*h>0*,*r>0*and*α<1*, the function*

$$P\left(\lambda \right)\;=\;\lambda^{3}{I^{*}}^{3}\left(1-I^{*}\right)+\lambda^{2}\left[{I^{*}}^{2}\left(1-I^{*}\right)\left(3\delta +2\gamma_{L}+r+f\right)-{I^{*}}^{3}\frac{r}{1-\alpha }\right]+\lambda \left[I^{*}\left(1-I^{*}\right)\left[\left(\delta +\gamma_{L}+f\right)\left(\delta +\gamma_{L}+r\right)+\delta \left(2\delta +2\gamma_{L}+r+f\right)\right]-{I^{*}}^{2}\frac{r}{1-\alpha }\left(2\delta +2\gamma_{L}+r+\alpha \beta f\right)\right]+\left(\delta +\gamma_{L}+r\right)\left(\delta \left(1-I^{*}\right)\left(\delta +\gamma_{L}+f\right)-\frac{rI^{*}}{1-\alpha }\left(\delta +\gamma_{L}+\left(1-\alpha \left(1-\beta \right)\right)f\right)\right)+fr\left(\delta +r\right)I^{*}$$

*has a non-negative root if and only if the constant term is non-positive, i.e.* 

(19) $$\left(\delta +\gamma_{L}+r\right)\left(\delta \left(1-I^{*}\right)\left(\delta +\gamma_{L}+f\right)-\frac{rI^{*}}{1-\alpha }\left(\delta +\gamma_{L}+\left(1-\alpha \left(1-\beta \right)\right)f\right)\right)\;+\;fr\left(\delta +r\right)I^{*}\leq 0.$$

*It has a strictly positive root if and only if one of the following conditions is met:*

*1.* *The constant term is strictly negative.*
*2.* *The constant term is*0*and the coefficient of the linear term is strictly negative, i.e.* 
  $$I^{*}\left(1-I^{*}\right)\left[\left(\delta +\gamma_{L}+f\right)\left(\delta +\gamma_{L}+r\right)+\delta \left(2\delta +2\gamma_{L}+r+f\right)\right]\;-\;{I^{*}}^{2}\frac{r}{1-\alpha }\left(2\delta +2\gamma_{L}+r+\alpha \beta f\right)<0.$$

**Fig. 2:**
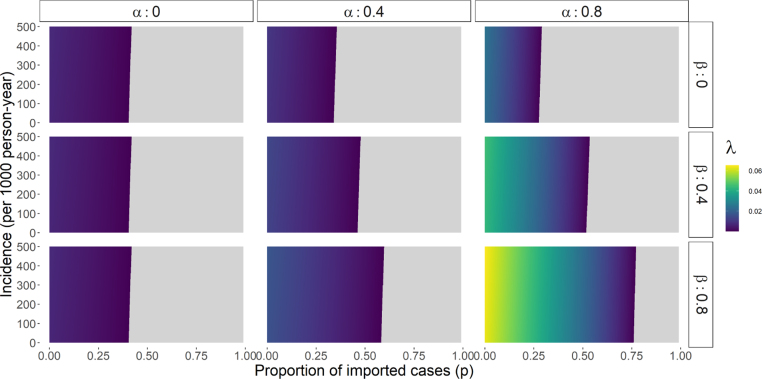
Calculated λ values for varying incidence and importation levels, under 9 case management scenarios (α and β). Grey areas indicate that there is no real positive solution for λ. Other parameters are fixed to the following values from [7] (f=1/72, $\gamma_{L}=1/223$, r=1/60) and ρ=1.

The proof is given in Appendix A. As an example, the parameter space for which λ is real positive is explored numerically in Fig. 2. Overall, λ is well defined when the proportion of imports does not exceed a certain threshold that depends strongly on case management parameters, and to a lesser extent on incidence.

Additionally, we need $I^{*}<1$ and therefore, $h<\frac{\rho r}{1-\alpha }$, leading to an upper bound on the feasible incidence space which is rarely reached in practical applications. In any case, it can be noted that the simplified representation of *P. vivax* transmission in this model is not suitable for high incidence settings, where immunity is expected to play a large role in the disease dynamics and should be explicitly modelled. Settings with important immunity levels could be characterized by an entomological inoculation rate over 10 and hence a parasite prevalence rate (measured by microscopy) approximately superior to 5% following results from [14].

## 4 Effect of the parameters on the model outcomes

In this section, we explore the sensitivity of the model outcomes depending on the inputs parameters. Because the model is intended to be used for an unknown transmission rate λ but with known incidence and importation levels, the analysis is conducted under three observed incidence scenarios (5, 50 and 100 cases per 1000 person–year) and two importation scenarios (0% and 10% of the new cases including both new infections and relapses). The model outcomes considered are the reproduction numbers ($R_{0}$ and $R_{c}$) and the proportion of new infections due to relapses (defined as $\frac{\rho fS_{L}^{*}}{h}$). Therefore, for each parameter set and scenario, the associated value for λ is calculated and used in the formulae given in (6), (5), (14). With this approach, we explore how from the same observed data different model outcomes can be inferred, based on various assumptions for input parameter values.

The six model parameters (r, $\gamma_{L}$, f, ρ, α and β) are sampled using a maximin latin hypercube sampling scheme of dimension 10,000 (from *lhs* R package [22]), with uniform ranges as indicated in Table 2. We neglect the dependence between the parameters governing the liver stage dynamics ($\gamma_{L}$ and f) [7], [23] and sample them independently to explore the effect of all their potential combinations on the outcomes.

**Table 2:** Parameter ranges considered in the LHS scheme for sensitivity analysis.

Parameter	Range	Reference
Biological parameters		
Blood stage clearance rate r (day^−1^)	1/85–1/35	centered around 1/60 [7]
Liver stage clearance rate $\gamma_{L}$ (day^−1^)	1/500–1/200	covering median values from [7]
Relapse frequency f (day^−1^)	1/175–1/40	covering median values from [7]

Case management parameters		
Proportion of notified cases ρ	0.1–0.8	assumed
Proportion of effective care α	0.1–0.8	assumed
Proportion of radical cure β	0–1	.

### 4.1 Uncertainty analysis

**Fig. 3:**
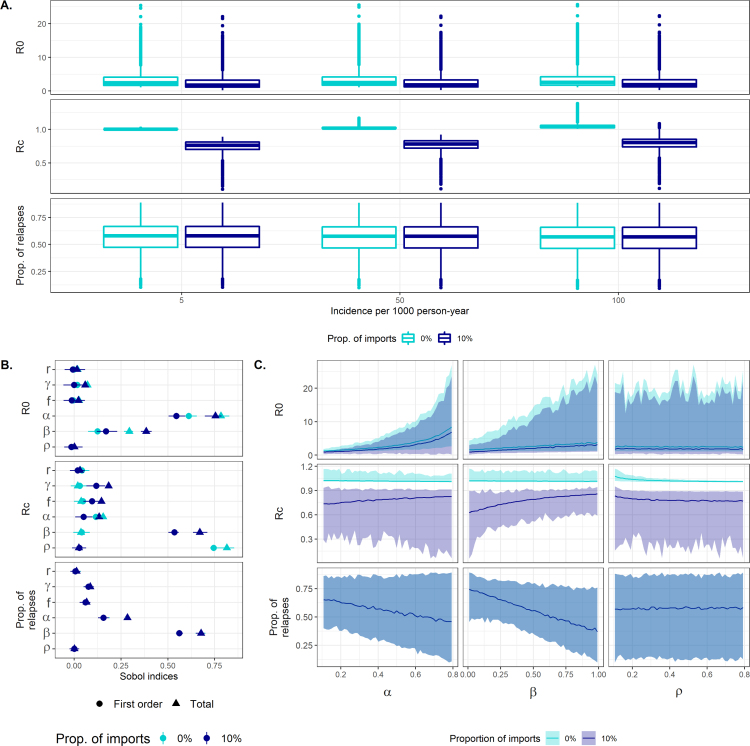
Sensitivity of $R_{0}$, $R_{c}$ and the proportion of relapses to the model parameters for differing incidence and importation scenarios. A. Distribution of the outcome values given the variability in parameters. B. Sobol indices (first order and total effects) quantifying the relative contribution of the input parameters to the variance in each outcome. Similar findings were observed for the 3 considered incidence levels, and only the scenario with incidence = 50 per 1000 PY is displayed. C. Relationship between the case management parameters and the three outcome variables. The simulated parameter ranges were subdivided into 50 bins, and the median, minimum and maximum within each bin are displayed (solid line and shaded area). Similar findings were observed for the 3 considered incidence levels, and only the scenario with incidence = 50 per 1000 PY is displayed.

The overall variation in the three outcome quantities is presented in Fig. 3A. $R_{0}$ values display large variability, with most values concentrated between 1 and 5, but ranging to more than 20 for some parameter sets. $R_{0}$ is lower when the importation rate is higher, but for the considered incidence and importation scenarios, the variation across parameter values within a given scenario is larger than the variation between these scenarios.

On the contrary, the importation level strongly influences the $R_{c}$ values. In the absence of importation, due to the model assumption of endemic equilibrium, $R_{c}$ values must be above 1. Consequently, the obtained values are very close to 1 and increase with increasing incidence. When importation is assumed to be 10%, $R_{c}$ values are largely variable between 0 and 1, with some values above 1 and most values close to 0.7. As expected, accounting for importation influences the qualitative interpretation of the reproduction number under control: the same incidence level corresponds to a higher local risk if it is entirely attributed to local transmission rather than if it is ignited by importation.

The proportion of relapses among new infections varies widely across parameters values, with a median close to 60%, and similar ranges of values are observed for the three incidence levels and the two importation levels.

### 4.2 Sensitivity analysis

The relative contribution of the parameters to the variability observed is explored by variance decomposition [24], calculating Sobol indices with the *soboljansen* function of the *sensitivity* R package [25], which is based on [26], [27]. The first order and total effects are presented in Fig. 3B. Overall, the parameters related to case management and reporting are more influential on the reproduction numbers and relapse proportion than the biological parameters governing the clearance and relapse rates. The shape of the association of the case management parameters with the three outcomes is displayed in Fig. 3C.

The effective coverage parameters (α and β) are the most influential on $R_{0}$ (Sobol indices above 0.6 and 0.2 respectively) and they are both positively associated with the outcome regardless of the importation level. Although these parameters do not enter in Eq. (6), their values influence the estimated transmission rate λ for a given observed incidence. We can interpret this association in the following way: when assuming high intensity of effective control, the only possibility to retrieve the observed incidence is with a higher intrinsic risk of transmission. And inversely, for the same observed incidence, a low intensity of control will suggest that the intrinsic risk is also low.

On the contrary, the effect of the model parameters on $R_{c}$ differs depending on the level of importation. In the absence of importation, the most influential parameter is the observation rate ρ (negative association): a higher observation rate indicates a lower number of unreported infections for the same number of reported infections and therefore a lower overall transmission risk. When importation is assumed to be 10%, the most influential parameter is the probability of radical cure β (positive association). Higher values for β lead to higher values of the transmission rate λ that overcompensate the direct negative effect of β on $R_{c}$.

Concerning the proportion of relapses, unsuprisingly, the probability of radical cure β is the most influential parameter: improving the treatment of liver-stage parasite reduces the proportion of relapses. This effect is very similar for the three incidence levels and the two importation levels considered.

## 5 Comparison of intervention effect sizes with those by [15]

One intended use of this model is to quantify the impact of anti-malarial interventions such as changes in case management. In the absence of a dataset with enough details for a formal validation, the model is confronted to a more detailed individual-based *P. vivax* transmission model [14], [15]. Because of their very different structure and levels of complexity, this individual-based model and the compartmental model represented by Eqs. (1)–(4) do not share the same scope of applications and are not interchangeable; however, it is important to ensure that they provide coherent results with one another. The objective is therefore to evaluate if the simple model presented here predicts intervention effect sizes of similar magnitude as those produced by [15] when increasing the proportion of radical cure by introducing tafenoquine in the treatment pathway. The parameters for the main scenario under the three incidence levels detailed in [15] were extracted and incorporated in the present model, as indicated in Table 3. For each setting, we first calculated the transmission parameter λ from the observed incidence under the primaquine scenario (PQ, using $\beta =\beta_{PQ}$). The associated equilibrium values for the state variables are also derived. These values are then used to simulate another scenario where the rate of radical cure was increased from $\beta_{PQ}$ to $\beta_{TQ}$. The observed incidence after 5 years of tafenoquine use is compared to the initial one to produce the effect sizes.

The present model managed to reproduce effect sizes of similar magnitude in the first two transmission settings but failed in the very high incidence setting (cf. Table 3). This is an encouraging finding given the large difference in model complexity. The discrepancy between the two models in the very high incidence setting can be explained by the strong role of immunity which is ignored in our simple model. Therefore, our simple model is not suitable for representing high transmission settings but provides reasonable results in low transmission settings where it is intended to be used.

**Table 3:** Comparison of effect sizes after 5 years in the model with those from [15]. *the blood stage clearance rate is not directly comparable with the model used in [15], therefore the baseline value from [7] was used.

Setting	Low incidence	Intermediate incidence	Very high incidence
Cases per 1000 PY	23	112	267
Assumed importation	0	0	0

Parameters from [15]			
Blood stage clearance rate* r (day^−1^)	1/60	1/60	1/60
Liver stage clearance rate $\gamma_{L}$ (day^−1^)	1/383	1/383	1/383
Relapse frequency f (day^−1^)	1/69	1/69	1/69
Proportion of clinical cases (assumed equal to ρ)	0.18	0.13	0.08
Proportion of effective care α	0.95ρ	0.95ρ	0.95ρ
Proportion of radical cure with PQ $\beta_{PQ}$	0.431	0.429	0.422
Proportion of radical cure with TQ $\beta_{TQ}$	0.59	0.605	0.619

Effect sizes			
In [15] (S1)	31.9% (19.8–44.3)	17.9% (13.2–21.7)	9.1% (6.8–10.4)
Calculated with the model	32%	17%	1%

## 6 Application: calculation of local reproduction numbers in Panama

The model is now applied to identify *P. vivax* transmission foci in Panama, by calculating local reproduction numbers based on the reported incidence from 2018. Like many countries in Central America, Panama is on the path to malaria elimination and its malaria burden is largely attributable to *P. vivax*. In 2018, more than 700 *P. vivax* cases were reported in the country (and only 2 *P. falciparum* cases), a total that was similar to previous years [28]. The identification of areas where transmission is sustained, as opposed to areas where malaria is importation-driven, could be helpful for targeting control interventions.

### 6.1 Data description

**Fig. 4:**
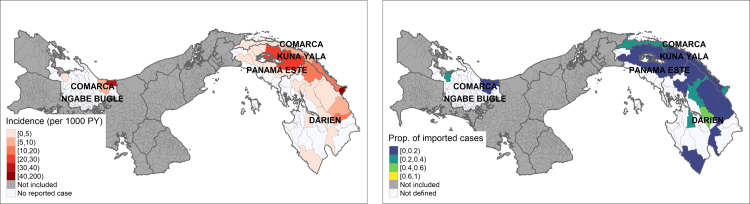
Case and importation *P. vivax* data in 2018 in all corregimientos within Panama’s four endemic health regions reporting at least one local case. Left panel: Incidence per 1000 person–year, missing values indicate the absence of any case report and therefore the likely absence of malaria transmission. Right panel: Proportion of the reported cases that originated from another *corregimiento*.

The spatial unit of the analysis are Panama’s *corregimientos*, the smallest administrative subdivisions above locality. Data on malaria cases were extracted from the malaria module of SISVIG, the Ministry of Health’s national surveillance system [29], with population denominators from the 2010 census [30]. In this database, each case is associated with a locality of detection i.e. where the malaria test was sampled, and a locality of origin i.e. where malaria transmission is considered to have occurred. The locality of origin is assigned manually for each case, based on the patient’s place of residence (default) and their history of travel in the last 30 days (if reported and available). For this analysis, the reported *P. vivax* cases during 2018 and population estimates per *corregimiento* were combined to calculate incidence at the *corregimiento* level (*corregimiento* of detection). We defined imported cases as either cases imported from overseas or cases where the *corregimiento* of origin was different from the *corregimiento* of detection. The incidence and proportion of imported cases per *corregimiento* are presented in Fig. 4. The analysis was restricted to *corregimientos* in the four malaria endemic health regions of the country (Darién, Kuna Yala, Ngäbe-Buglé and Panamá Este) where at least one local case was observed. In the areas considered, reported incidence in 2018 ranged from 0 to 109 cases per 1000 person–year with varying proportions of importation between 0 and 50%.

### 6.2 Statistical model for representing data uncertainty

In order to propagate data uncertainty in the model outcomes, the uncertainty on the measurement of h and p can be modelled as follows.

Let c be the annually reported number of cases in a given area with population size N. We can assume that c is a random draw from the Poisson-distributed variable $C∼P\left(\overset{\sim }{h}N\right)$, where $\overset{\sim }{h}$ is the annual rate of reported incidence (using $\overset{\sim }{h}=365h$). We can perform inference on $\overset{\sim }{h}$ in the Bayesian framework, using the conjugate Jeffreys prior $\overset{\sim }{h}∼Gamma\left(1/2,0\right)$, thus giving the following posterior distribution (as for example in [31]):

$$\overset{\sim }{h}|\left(C=c\right)∼Gamma\left(1/2+c,N\right)$$

Similarly, let y be the annually reported number of imported cases in a given area with c annually reported cases. We can assume that y is a random draw from the binomial-distributed variable Y∼B(p,c), where p is the probability that a case is imported. We can perform inference on p in the Bayesian framework, using the conjugate Jeffreys prior p∼Beta(1/2,1/2)), thus giving the following posterior distribution:

p|(Y=y)∼Beta(1/2+y,1/2+c−y)

**Fig. B.7:**
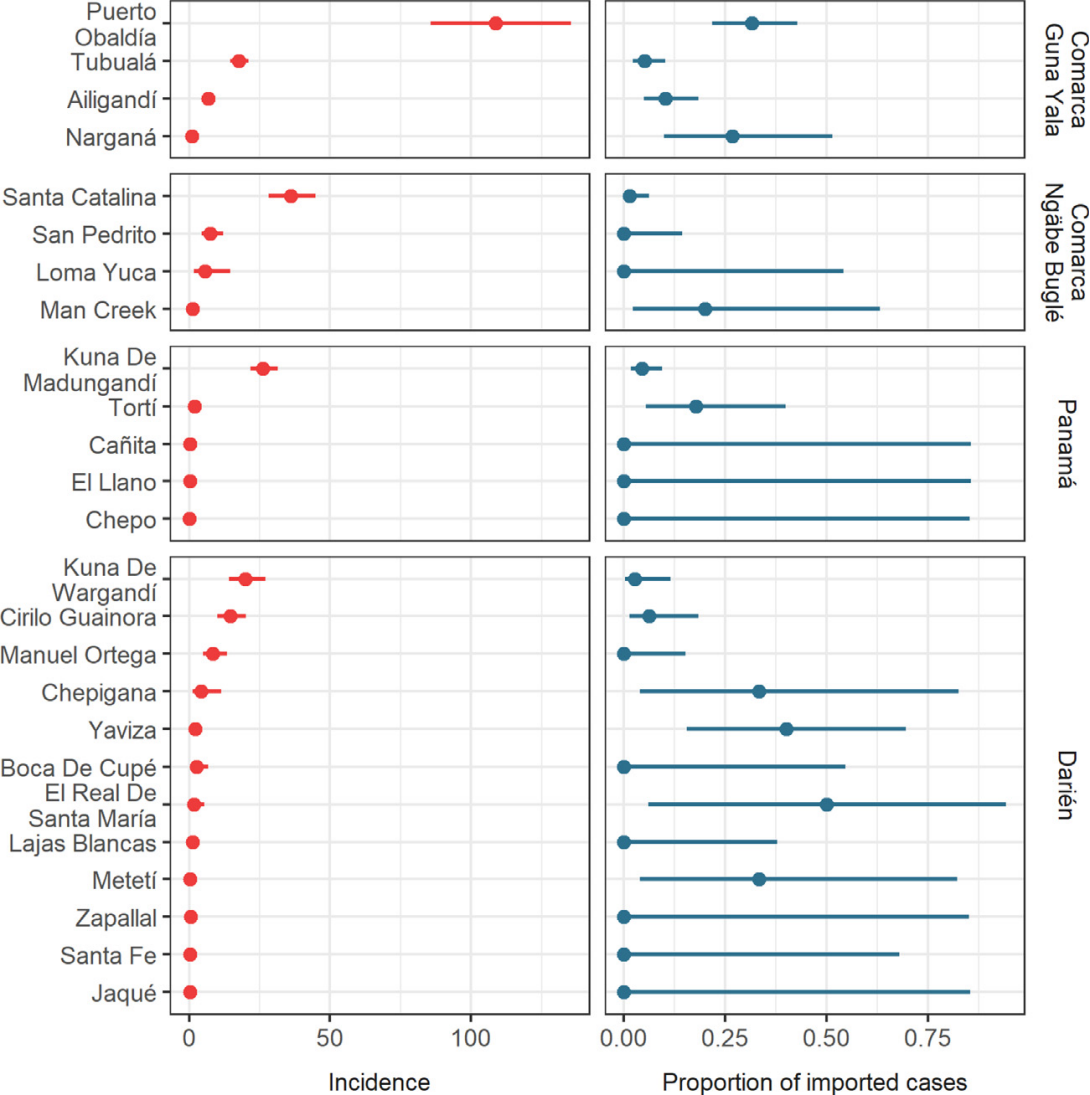
Credible intervals for the annual incidence (per 1000 person–year) and p per *corregimiento*, based on 10,000 random draws from their posterior distributions. Only *corregimientos* within the four endemic health regions reporting at least one local case in 2018 and where the validity conditions are met for the observed values p and $\overset{\sim }{h}$ are displayed. The dots indicate the observed values for the incidence and p.

For each *corregimiento*, 10,000 sets for $\left(p,\overset{\sim }{h}\right)$ were drawn from these two posterior distributions, and the resulting credible intervals are shown in Fig. B.7 in B. By construction, when no imported case was reported, the observed value for the proportion of importation lies at the edge of the credible interval. The corresponding $R_{0}$ and $R_{c}$ can then be derived for each set, thus providing uncertainty estimates on these quantities.

### 6.3 Model parameterization

The biological parameters (r, f and $\gamma_{L}$) are fixed as in [7] whereas the case management parameters are selected to reflect the Panama context. We assumed that all reported cases are treated with anti-schizonticidal drugs (ρ=α). This assumes that all cases diagnosed with a *P. vivax* infection are reported in the system and receive a chloroquine treatment with 100% efficacy against the blood stage parasites. These cases are assumed to be those who simultaneously 1) present clinical symptoms (assuming they do not have low parasite densities and represent approximately 30% of the infections [32]), 2) seek treatment with rate τ and 3) are diagnosed positive with a rapid diagnostic test with 95% sensitivity [33]. The treatment seeking rate τ is highly uncertain, and therefore, 3 scenarios were considered (25, 50 and 75%), leading to effective cure rates α of 7, 14 and 21%.

The probability of liver stage clearance with PQ (β) was assumed to depend on adherence and drug efficacy. Adherence was assumed to be 100% for the individuals receiving Directly Observed Therapy (DOT), i.e. 64.5% of the cases. For those that did not receive DOT, we estimate that 62.2% of them would complete treatment, based on an estimate from Peru [34] with use of the same PQ scheme. This amounted to an additional 21.8% of patients completing treatment. A study in Colombia with the same PQ scheme [35] has been found to result in 18% recurrence rate for patients completing treatment and we use this value to evaluate the treatment failure probability. For patients completing the PQ treatment, relapses occur with a probability equal to the product of the treatment failure probability and the baseline relapse probability. The baseline relapse probability is $f/\left(f+\gamma_{L}\right)$, as some individuals might clear their liver-stage parasites before relapsing, and it is approximately equal to 0.76 with the chosen parameter values. Thus, we assume the PQ treatment failure probability to be equal to 0.18/0.76 = 0.24, corresponding to 76% PQ efficacy. With these assumptions, it is assumed that 66% (0.76 * (64.5 + 21.8)) of treated individuals successfully clear their liver-stage parasites. We assume that there is no testing for G6PD deficiency, and that individuals are not excluded for this purpose.

One *corregimiento* fell outside of the validity condition space for positive λ: it has an incidence equal to 1.7 per 1000 PY with 50% of imported cases, and was excluded from the subsequent analyses.

### 6.4 Results

**Fig. 5:**
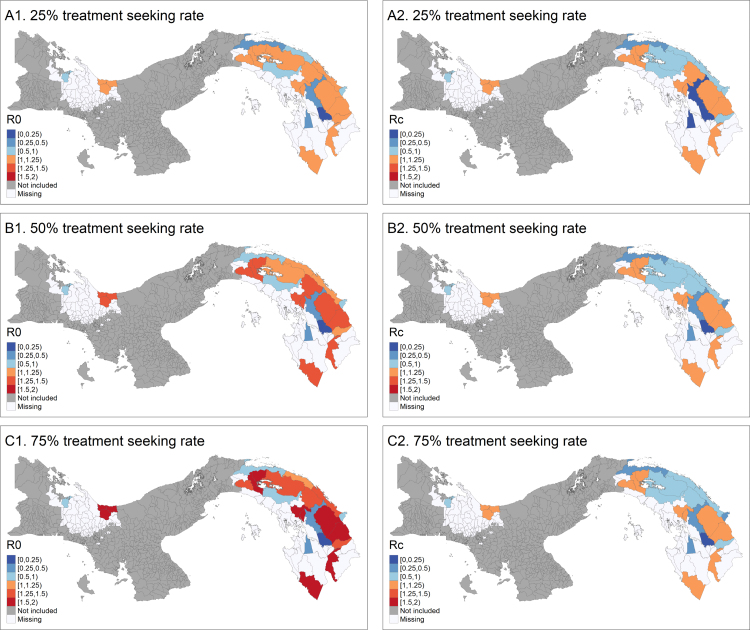
Reproduction numbers calculated by the model - $R_{0}$ and $R_{c}$ - for three case management scenarios. A1, B1 and C1 display the basic reproduction number $R_{0}$ while A2, B2 and C2 display the reproduction number in the presence of control $R_{c}$. A. Assuming that treatment seeking rate is 25%, α=ρ=0.07. B. Assuming that treatment seeking rate is 50%, α=ρ=0.14. C. Assuming that treatment seeking rate is 75%, α=ρ=0.21. The other parameters are fixed as follow: r=1/60, f=1/72, $\gamma_{L}=1/223$ [7], and β=0.66.

The estimated local reproduction numbers with and without control are presented in Fig. 5. The model can identify *corregimientos* where both $R_{0}$ and $R_{c}$ are below the threshold value 1, indicating that malaria transmission in these regions is only sustained through importation, and would stop if the area was isolated. In other *corregimientos*, $R_{0}$ is above 1 but $R_{c}$ is below 1, indicating that current case management practice keeps transmission under control, and malaria propagation would stop in the absence of importation if interventions are maintained at their current level. Finally, in a certain number of *corregimientos*, both $R_{0}$ and $R_{c}$ are estimated above 1, indicating ongoing malaria transmission and a potential need for additional control interventions, however, it is important to note that $R_{c}>1$ by construction in all areas where the proportion of importation is zero. Assuming higher access to care leads generally to higher $R_{0}$ values and lower $R_{c}$ values.

**Fig. 6:**
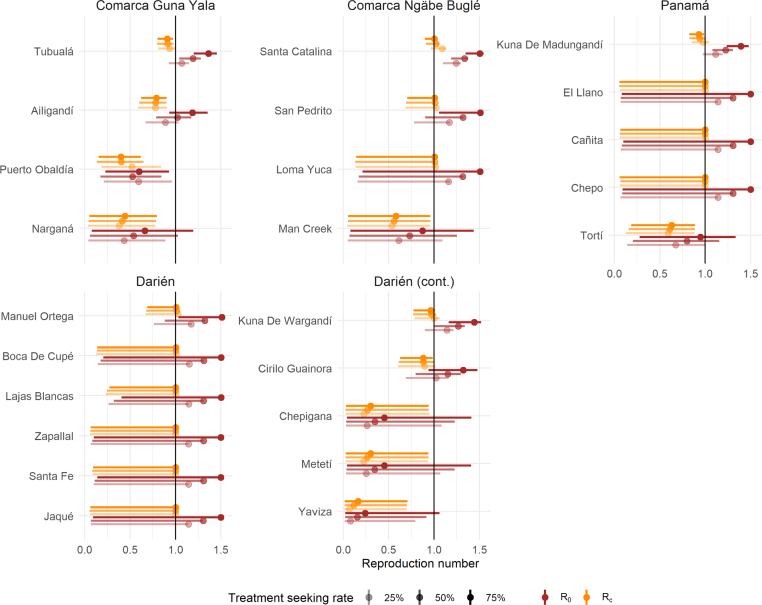
Reproduction numbers and associated modelled uncertainty, in the different *corregimientos* grouped by health region (based on 10,000 draws). Only *corregimientos* within the four endemic regions reporting at least one local case in 2018 and where the validity conditions are met for the observed values p and $\overset{\sim }{h}$ are displayed. The dots indicate the point estimate obtained for the observed values of $\overset{\sim }{h}$ and p. The error bars indicate the 95% credible intervals from the empirical distribution of $R_{0}$ and $R_{c}$ values obtained from the 10,000 draws from the posterior distribution of $\overset{\sim }{h}$ and p. The black vertical line indicates the threshold value 1. Some the posterior draws from p and $\overset{\sim }{h}$ lead either to invalid parameter combinations for a positive λ or to h values close to zero (the selected cut-off was $h<1.10^{-8}$): in these cases, the reproduction numbers could not be calculated, and these draws are ignored in the credible interval calculation.

When accounting for the uncertainty in data observation (cf. Fig. 6), in some *corregimientos* where the $R_{c}$ point estimate was below 1, the credible interval for $R_{c}$ contains the value 1 and hence the presence of sustained transmission cannot be ruled out. Conversely, in all areas where the $R_{c}$ point estimate was above 1, the credible interval extends below the value 1: there is therefore a relevant amount of uncertainty in the data that needs to be accounted for when interpreting the obtained results. The uncertainty is particularly large in *corregimientos* with very few reported cases and no reported importation (and hence where the $R_{c}$ point estimate is above 1 by construction). In these settings, the point estimate for both reproduction numbers lies on the edge of their credible interval because all posterior draws include a larger amount of importation than the observed data, leading to smaller reproduction numbers. Overall, of the 24 *corregimientos* in the four endemic regions that have observed local incidence and for which the validity conditions were verified, 15 have credible intervals for $R_{c}$ that contain the value 1 for at least one treatment seeking scenario: there is thus a potential for active transmission in these areas and hence a need for additional or strengthened interventions. Nevertheless, given the results of the uncertainty analysis and the variability across treatment seeking scenarios, a finer quantification of access to care probabilities would be required in order to draw actionable conclusions in the Panama context, which is beyond the scope of the current manuscript.

## 7 Discussion and conclusion

This work builds on previous literature [7] and presents a parsimonious compartmental model for *P. vivax* transmission that includes imported infections and case management control interventions. It also highlights how to calculate the transmission rate associated with a certain observed incidence by solving the steady state of the model. The simplicity of this model makes it a desirable tool to answer programmatic questions in a rapid and transparent manner. This is illustrated with an application of the model to calculate local reproduction numbers in Panama: the model can identify areas of sustained transmission that can be targeted for additional or strengthened control interventions. The model can also be used to simulate the effect of changes in the intervention strategies as it manages to produce effect sizes of similar magnitude as a more complex individual-based model [15] for settings with low and moderate transmission intensities.

Although the mosquito dynamics are not explicitly modelled, vector control can be directly included in the model as a reduction of the intensity of transmission λ, following [36]. The transmission parameter λ becomes ωλ where ω∈[0,1] represents the intensity of vector control and can be informed by an external model for vector dynamics [36], [37]. The absence of vector control corresponds to the case ω=1, and ω=0 represents perfect vector control that completely disables vector-borne transmission. The methodology presented in Section 3 remains unchanged, except that it calculates ωλ and not λ. This change would only influence the intrinsic risk $R_{0}$ (values $R_{c}$ remaining unchanged) and the extended model could also be used for simulation of vector control intervention scenarios.

Thanks to its simple compartmental structure and the availability of analytical results, the model provides almost instantaneous computation of reproduction numbers, and rapid simulation of model dynamics. Therefore, uncertainty in the data or in parameter distributions can be very easily propagated in the estimates and simulations when they are available. One example of uncertainty quantification for incidence and importation data was presented here, but other statistical models could be used similarly, to better reflect the assumptions in the data generation process.

The simplicity in the compartmental model’s structure also makes it possible to perform sensitivity analyses over a large sampling space to explore the influence of parameters on the outputs. In particular, we show that this model is most sensitive to parameters reflecting the intensity of case management (effective cure rate of blood and liver stages as well as reporting), thus highlighting the importance of precise measures of these parameters or of an appropriate quantification of their uncertainties. With additional data on regional differences in case reporting and case management access in Panama, the application presented here can be extended to better account for local variations in current case management practices when identifying transmission foci.

The degree of case importation also had a strong influence on the model result: as expected, the local transmission is higher if all the reported cases contracted the disease locally rather than elsewhere. Therefore, patient travel history provides valuable information in order to disentangle the relative roles of autochthonous transmission and importation on the observed incidence patterns, even when the origin of transmission is not fully known [38]. Notably, this method only accounts for returning travellers and not for individuals from other areas that would initiate local transmission chains (visitors) [39] and this simplification could lead to an overestimation of local transmission potential if the amount of visitors is large. We also assume that imported and local infections are reported with the same rate and that there is no systematic bias in reporting between the two. Nevertheless, with this modelling choice, local importation patterns can be accounted for in a simplified way, which can prove very useful when the detailed mobility data necessary to build an inter-connected model [40] is not available.

This methodology has also some limitations. First of all, due to its simplicity, this model ignores several biological mechanisms of parasite propagation. In particular, the development of immunity is not included because it is assumed to play a minor role in the low transmission settings where this model is intended to be used. In areas with high transmission intensity, other more complex models accounting for immunity should be used instead. The effect of seasonality was also ignored, as only annual data was used. Therefore, the model might underestimate the risk in the presence of very short seasonal transmission peaks: it can nevertheless help to identify “relative spatial differences in risk across the study area” [41]. Importantly, case management is included in the model in a simplified way, assuming that individuals manage to be cured before they are capable of transmitting the disease. This is an important simplification, as symptomatic patients with *P. vivax* infections often carry mature gametocytes at the time of treatment [42]. This model may therefore overestimate the impact of case management on *P. vivax* transmission. Further extensions of the model including an interval before treatment during which transmission can occur will be considered in future work.

Secondly, the calculation of the transmission rate using observed incidence relies on the steady-state assumption. Although it provides a simple expression for model calibration, it makes the assumption that transmission is stable over the years, and that transmission would remain at the same constant level in the absence of any intervention changes. If sufficient data is available to study the temporal trend in reported incidence, other statistical methods for fitting [43] could be used to relax this assumption. Moreover, as countries get closer to elimination and reach very low incidence levels, the steady-state assumption is unlikely to hold at local levels and other models could be used, either by considering connectivity across all areas and assuming global equilibrium only [40], [44] or by using other approaches for foci detection in very low transmission settings (less than 20 reported cases per year) similar to [45].

Finally, the model is deterministic: it does not reproduce the variability observed in very low transmission settings or for very small population sizes and therefore cannot simulate elimination events. Nevertheless, the simplicity of the compartmental structure enables direct extensions of the model for stochastic simulations using the Gillespie or τ-leap algorithms. With the stochastic version of this model, the probability of elimination under several intervention scenarios could be computed.

Despite these limitations, this model has the advantage that it requires very short running times, and that analytical results are available for its calibration at equilibrium and the conditions thereof. These advantages also make it a portable tool that could be used by a wide range of users, without specific computer language or code requirements. In order to enhance country-specific applications, an R package for reproduction number calculation and simulations with this model has been created (https://github.com/SwissTPH/VivaxModelR).

In conclusion, we present a model of *P. vivax* dynamics which is suitable for an intermediate range of transmission settings and whose flexibility and transparency are useful for timely country-specific applications.

## CRediT authorship contribution statement

**Clara Champagne:** Conceptualization, Methodology, Software, Formal analysis, Investigation, Writing – original draft. **Maximilian Gerhards:** Conceptualization, Methodology, Formal analysis, Investigation, Writing – original draft. **Justin Lana:** Resources, Investigation, Writing – review & editing, Project administration. **Bernardo García Espinosa:** Resources, Data Curation, Investigation, Writing – review & editing, Project administration. **Christina Bradley:** Resources, Writing – review & editing, Project administration. **Oscar González:** Resources, Data curation, Writing – review & editing. **Justin M. Cohen:** Conceptualization, Writing – review & editing, Project administration, Funding acquisition. **Arnaud Le Menach:** Conceptualization, Writing – review & editing, Project administration, Funding acquisition. **Michael T. White:** Conceptualization, Writing – review & editing. **Emilie Pothin:** Conceptualization, Methodology, Formal analysis, Writing – review & editing, Project administration, Funding acquisition, Supervision.

## Declaration of Competing Interest

The authors declare that they have no known competing financial interests or personal relationships that could have appeared to influence the work reported in this paper.

## Data Availability

The computer code utilized in this manuscript is available at: https://github.com/SwissTPH/VivaxModelR. The malaria case data is property of the Ministry of Health of Panama and cannot be made publicly available at this resolution to respect privacy.

## Appendix A Proofs

Proof of Theorem 1 Before beginning the proof, we point out the fact that h>0, r>0 and α<1 imply $0<I^{*}<1$. To make the dependence of P on f visible, let us denote it $P_{f}$. Then,

$$P_{f}\left(\lambda \right)=P_{0}\left(\lambda \right)+fQ\left(\lambda \right)$$

with

$$P_{0}\left(\lambda \right)=\left(\lambda I^{*}+\delta +\gamma_{L}\right)\left(\lambda I^{*}+\delta +\gamma_{L}+r\right)\left(\left(\lambda I^{*}+\delta \right)\left(1-I^{*}\right)-\frac{r}{1-\alpha }I^{*}\right)$$

and

$$Q\left(\lambda \right)=\lambda^{2}{I^{*}}^{2}\left(1-I^{*}\right)+\;\lambda \left[I^{*}\left(1-I^{*}\right)\left(\left(\delta +\gamma_{L}+r\right)+\delta \right)-{I^{*}}^{2}\frac{\alpha \beta }{1-\alpha }r\right]+\;\left(\delta +\gamma_{L}+r\right)\left(\left(1-I^{*}\right)\delta -I^{*}\frac{1-\alpha \left(1-\beta \right)}{1-\alpha }r\right)+r\left(\delta +r\right)I^{*}=\underset{R\left(\lambda \right)}{\underset{︸}{\left(\lambda I^{*}+\delta +\gamma_{L}+r\right)\left(\left(\lambda I^{*}+\delta \right)\left(1-I^{*}\right)-\frac{\alpha \beta }{1-\alpha }rI^{*}\right)}}-\gamma_{L}rI^{*}.$$

We will start with the case f=0. From the shape of $P_{0}$ one can easily see that the three roots in that case are

$$-\frac{\delta +\gamma_{L}+r}{I^{*}},\;-\frac{\delta +\gamma_{L}}{I^{*}}\;\text{and}\;\frac{r}{1-\alpha }\frac{1}{1-I^{*}}-\frac{\delta }{I^{*}}.$$

ordered from smallest to largest. Only the last two of them may be non-negative, and the middle one only in the case $\delta =\gamma_{L}=0$, and in that case, from λ=δ=f=0 it follows h=0, contradicting our assumption, so this is no solution. Now we turn to the case f>0. It is easily seen that $P_{0}+fR$ is a polynomial of degree 3 with positive leading coefficient and a root at $\lambda_{1}≔-\frac{\delta +\gamma_{L}+r}{I^{*}}$. Let us now first consider the case $\gamma_{L}>0$. In that case we also see that

(A.1) $$\underset{<0}{\underset{︸}{P_{0}\left(-\frac{\delta }{I^{*}}\right)}}+\underset{\leq 0}{\underset{︸}{fR\left(-\frac{\delta }{I^{*}}\right)}}<0.$$

As $\lim_{\lambda \to \infty }P_{0}\left(\lambda \right)+fR\left(\lambda \right)=\infty$, from the intermediate value theorem it follows that $P_{0}+fR$ has a root $\lambda_{3}$ to the right of $-\frac{\delta }{I^{*}}$. Since every simple root is accompanied with a sign change, the last root $\lambda_{2}$ has to be to the left of $-\frac{\delta }{I^{*}}$. In particular, $\lambda_{1}$ and $\lambda_{2}$ are both negative. We will now prove that by adding $-f\gamma_{L}rI^{*}$, we will not get a non-negative root: Since $-f\gamma_{L}rI^{*}<0$, we have

(A.2) $$P_{f}\left(\lambda \right)=P_{0}\left(\lambda \right)+fR\left(\lambda \right)-f\gamma_{L}rI^{*}<0\;\text{for }\lambda \in \left[0,\lambda_{3}\right],$$

implying that there cannot be a root in that interval. On the other hand, it is a known fact that the inflection point of a polynomial of degree 3 with three real roots lies between the smallest and the largest root. This implies that $P_{0}+fR$ is strictly convex in the interval $]\lambda_{3},\infty [$. Since $P_{f}=P_{0}+fR-f\gamma_{L}rI^{*}$ has the same second derivative, it is also strictly convex in that interval. This, combined with the fact that $P_{f}\left(\lambda_{3}\right)=-f\gamma_{L}rI^{*}<0$, implies that there is only one root of $P_{f}$ in that interval. This finishes the proof in the case $\gamma_{L}>0$. Now we turn to the case $\gamma_{L}=0$. Then, $P_{f}=P_{0}+fR$. We again know the root $-\frac{\delta +r}{I^{*}}$ and we see that

(A.3) $$P_{f}\left(-\frac{\delta }{I^{*}}\right)=\underset{=0}{\underset{︸}{P_{0}\left(-\frac{\delta }{I^{*}}\right)}}+fR\left(-\frac{\delta }{I^{*}}\right)=-\underset{\neq 0}{\underset{︸}{fr}}\frac{\alpha \beta }{1-\alpha }\underset{\neq 0}{\underset{︸}{rI^{*}}}.$$

If this term is 0 (which is the case if and only if αβ=0), we see that $-\frac{\delta }{I^{*}}$ is also a root of $P_{f}$. This root is only non-negative if δ=0, which is the case of endless relapses and recoveries without liver-stage clearance. Otherwise, the term must be negative. Then again, since every simple root is accompanied with a sign change, we know that there must be exactly one root to the right of $-\frac{\delta }{I^{*}}$. □

Proof of Theorem 2 These are direct consequences of the fact that the polynomial function P has positive leading coefficient and at most one positive root: The leading coefficient being positive implies that $\lim_{\lambda \to \infty }P\left(\lambda \right)=+\infty$. The constant term is the value P(0). If this is negative, by the intermediate value theorem there has to be a root between 0 and ∞, i.e. a positive root. If P(0)>0, the fact that $\lim_{\lambda \to \infty }P\left(\lambda \right)>0$ implies that there has to be an even number of places the λ-axis is crossed between 0 and ∞. As there is only at most one single strictly positive root, there must be 0. The last case is P(0)=0. In that case we obviously found a non-negative root. For an answer to the question about an additional, strictly positive root, we look at the coefficient of the linear term. This is the value of $P^{\prime }\left(0\right)$. If $P^{\prime }\left(0\right)<0$, there exists a λ>0 with P(λ)<0, so with the same argument as above, there must be a positive root between this λ and ∞. In the same vein, if $P^{\prime }\left(0\right)>0$, for any sufficiently small λ>0, P(λ)>0. There cannot be a root larger than any such λ, as these crossings of the λ-axis would have to appear pairwise as above. The last case is $P^{\prime }\left(0\right)=0$. In that case, we already have two (coinciding) non-negative roots of P(λ) at 0. As we have ruled out the possibility of three non-negative roots, there cannot be any further. □

## Appendix B Additional results: uncertainty quantification on incidence and proportion of importation

See Fig. B.7.
